# Interictal burden, not ictal burden, drives work productivity impairment in headache disorders: insights from a Japanese cross-sectional study

**DOI:** 10.1186/s10194-026-02284-4

**Published:** 2026-02-03

**Authors:** Masahito Katsuki, Noboru Matsumoto, Yasuhiko Matsumori, Muneto Tatsumoto, Keisuke Suzuki, Tomokazu Shimazu, Miguel Á. Huerta, Daiki Sato, Koki Kikugawa, Shigeharu Kamado, Siobhán O’Connor, Tomás Ward, Kieran Moran

**Affiliations:** 1 Physical Education and Health Center, Nagaoka University of Technology, 1603-1, Kamitomiokamachi, Nagaoka, Niigata ZIP 9402188 Japan; 2 School of Health and Human Performance, Dublin City University, Dublin, Ireland; 3 Insight Research Ireland Centre for Data Analytics, Dublin City University, Dublin, Ireland; 4 Department of Biostatistics, Graduate School of Medicine, Saitama Medical University, Saitama, Japan; 5 Division of Psychology, Faculty of Arts, Shinshu University, Nagano, Japan; 6 Baycrest’s Rotman Research Institute, University of Toronto, Toronto, Canada; 7 Sendai Headache and Neurology Clinic, Sendai, Miyagi Japan; 8 Canon Marketing Japan Inc., Minato-ku, Tokyo, Japan; 9 Department of Neurology, Dokkyo Medical University, Mibu, Tochigi Japan; 10 Department of Neurology, Saitama Neuropsychiatric Institute, Saitama City, Saitama Japan; 11 Department of Pharmacology, University of Granada, Granada, Spain; 12 Biosanitary Research Institute ibs.GRANADA, Granada, Spain; 13 Department of Pharmacology, University of Cambridge, Cambridge, UK; 14 Tsubamesanjo Sugoro Neurospine Clinic, Sanjo, Niigata Japan; 15 Kikugawa Neurology Clinic, Tsubame, Niigata Japan; 16 Department of Mechanical Engineering, Nagaoka University of Technology, Nagaoka, Japan; 17 Department of Sport Science and Nutrition, Maynooth University, Kildare, Ireland; 18 Insight Research Ireland Centre for Data Analytics, Maynooth University, Kildare, Ireland

**Keywords:** Absenteeism, Activity impairment, Migraine, Presenteeism, Structural equation modelling (SEM), Tension-type headache (TTH), Work productivity

## Abstract

**Objective:**

Headache disorders cause work productivity and activity impairment (WPAI). There are two distinct types of burden caused by headache disorders: interictal burden measured by Migraine Interictal Burden Scale-4 (MIBS-4) and ictal burden measured by Headache Impact Test-6 (HIT-6). However, the impact of interictal burden on WPAI remains unclear. This study aimed to investigate whether MIBS-4 score (interictal burden) is associated with WPAI among individuals with headache disorders, in contrast to HIT-6 score (ictal burden).

**Methods:**

We conducted a school-based online survey of students’ parents in Tsubame City, Japan, in 2024. The questionnaire included age, sex, headache characteristics, MIBS-4 and HIT-6 scores, and overall work productivity impairment (OWPI) assessed using the WPAI questionnaire. A structural equation model (SEM) evaluated the effects of MIBS-4 and HIT-6 scores on OWPI. The headache diagnosis was solely based on the questionnaire.

**Results:**

Among 5,227 households, 21.6% (1,127) responded, and 678 responses from parents with headache disorders were analyzed (median age 43, IQR: 39–47 years; 92.9% female). Median MIBS-4 score was 4 (2–6), HIT-6 score 58 (53–64), and OWPI 20.0% (10.0% – 30.0%). Only MIBS-4 score was significantly associated with OWPI (standardized coefficients [β] = 0.23, 95% CI: [0.14–0.33]), whereas HIT-6 score was not (β = 0.06, [-0.03–0.16]). Younger age, female sex, longer duration of headache, more monthly headache days, moderate/severe pain, presence of nausea/vomiting, photophobia, phonophobia, and osmophobia were directly associated with MIBS-4 score, and these clinical characteristics indirectly associated OWPI mediated via MIBS-4 score, whereas HIT-6 was not.

**Conclusions:**

OWPI was associated with MIBS-4 score (interictal burden), not HIT-6 score (ictal burden). Additionally, clinical characteristics are indirectly associated with OWPI mediated via MIBS-4 score. The stronger link between MIBS-4 and WPAI may be due to the persistent nature of psychological symptoms during the interictal period, which imposes a daily burden. The gender imbalance in this study may limit the generalizability of the findings to male workers, suggesting that workplace interventions may need to consider gender-specific burdens. Nevertheless, to improve OWPI, greater emphasis should be placed on addressing interictal burden.

**Supplementary Information:**

The online version contains supplementary material available at 10.1186/s10194-026-02284-4.

## Graphical Abstract

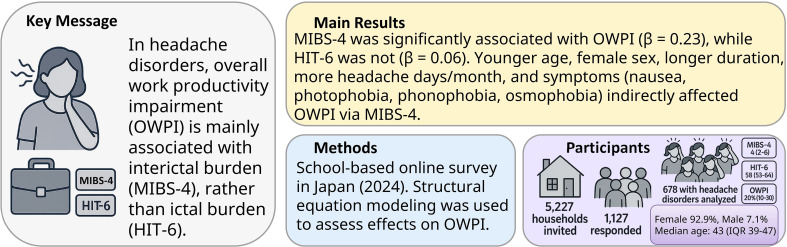

## Introduction

Japan has long been recognized for its long working hours and strong work ethic. However, despite these traits, Japan ranked 29th out of 38 Organisation for Economic Co-operation and Development (OECD) countries in work productivity, lagging behind other developed economies [1]. Most discussions around the causes of Japan’s low work productivity have focused on structural and institutional issues, including the high prevalence of small- and medium-sized enterprises, delayed digital transformation, rigid work practices [2], and the underutilization of women and older adults in the workforce [3]. While these factors are undoubtedly important, the impact of health conditions—particularly chronic, non-fatal disorders such as headache disorders—has recently begun to receive attention in discussions on work productivity [4].

Headache disorders, such as migraine, tension-type headache, and trigeminal autonomic cephalalgias, as classified by the International Classification of Headache Disorders, 3rd edition (ICHD-3) [5], are prevalent neurological conditions causing a significant global socioeconomic burden [6]. Headache disorders shorten healthy life expectancy, and rank as the second leading cause of disability-adjusted life-years among 15 neurological disorders, including stroke and dementia [7]. Furthermore, the Global Burden of Disease Study 2019, the last iteration before now, found migraine to be the second globally highest cause of years lived with disability, a measure of lost health attributed to non-fatal disease [8]. Headache disorders largely affect a wide range of age groups, mainly young and middle-aged females [8].

There is evidence that headache disorders negatively impact work productivity, as measured by the Work Productivity and Activity Impairment (WPAI) questionnaire with four subscales of loss [9]: WPAI assesses absenteeism, presenteeism, overall work productivity impairment (OWPI, calculated by absenteeism and presenteeism), and activity impairment [9]. Absenteeism refers to being absent from work altogether, whereas presenteeism refers to reduced productivity while working due to health problems [9, 10]. Patients with headache disorders, particularly those with migraine, exhibit substantial impairment on some subscales of the WPAI, ranging from 30% to 70% [9, 11–13]. Higher headache frequency [11, 14–16], severe headache intensity [14, 15], longer attack duration [16], higher frequency of analgesic use [16], female sex [17] or male sex [16], comorbid sleep disturbances [18], and depression [18] have been reported to worsen WPAI subscales further among patients with headache disorders. Presenteeism and OWPI, among the four WPAI subscales, have demonstrated good validity in studies involving patients with migraine [10]. Of the two, we focused on OWPI because it comprehensively captures both absenteeism and presenteeism as work productivity impairment.

In recent years, there has been a growing emphasis on assessing not only the ictal burden of headache disorders, but also the interictal psychological burden resulting from anticipatory anxiety, avoidance of activity, fatigue, difficulty with planning, and psychological stress during headache-free intervals [19–21]. This model suggests that headache disorders are conceptualized as a cycle in which the ictal and interictal periods alternate, and emphasizes the importance of focusing on and evaluating each period separately [20, 22, 23]. However, existing research has primarily focused on either the ictal or interictal burdens in isolation, without addressing the dual impact of both on work productivity.

The ictal and interictal headache-related burdens are assessed by two validated instruments: Headache Impact Test-6 (HIT-6) and Migraine Interictal Burden Scale-4 (MIBS-4), respectively [19]. HIT-6 quantifies the functional and symptomatic ictal burden experienced during headache episodes, assessing six domains such as pain intensity, limitations in daily activities, fatigue, and emotional strain, with scores ranging from 36 to 78 [24]. HIT-6 score has a correlation with WPAI [17]. Meanwhile, MIBS-4 quantifies the interictal psychological and anticipatory burden that occurs between headache episodes, including workplace or academic disruption, social and family life impairment, difficulty with planning, and emotional-cognitive distress. It comprises four items rated on a five-point scale, yielding a total score between 0 and 12 [25]. The higher the score on either scale, the greater the burden. However, interictal burden and the cyclical model with dual aspects are a recently proposed concept in 2009 [25], and the association between MIBS-4 score and OWPI, in contrast to HIT-6 score, remains unclear.

This study aimed to investigate whether MIBS-4 score (interictal burden) is associated with OWPI, one of the subscales of WPAI, in contrast to HIT-6 score (ictal burden). The second aim was to clarify whether MIBS-4 and HIT-6 scores mediate the associations between clinical characteristics of headache and OWPI. We conducted a school-based online survey among parents in a small Japanese city to collect data on individuals with headache disorders. We then statistically investigated the relationship between MIBS-4 score, OWPI, and other clinical characteristics, in contrast to HIT-6 score.

## Materials and methods

### Overall procedure

We conducted an online questionnaire survey targeting individuals with headaches to collect data on clinical headache characteristics, interictal and ictal burden (measured by MIBS-4 and HIT-6), and OWPI (measured by the WPAI questionnaire). To examine the relationship between MIBS-4 score and OWPI, we constructed a structural equation model (SEM). In the SEM, we defined clinical characteristics as initial exogenous variables, OWPI as outcome, as well as MIBS-4 and HIT-6 scores as mediators. We evaluated the association between MIBS-4 score and OWPI, as well as the indirect effects of clinical characteristics on OWPI via MIBS-4 score. Finally, to test the robustness of our findings, we performed sensitivity analyses using multivariable regression models.

### Online questionnaire procedure

To obtain the data on individuals with headache disorders, an online survey was carried out between September and December 2024 in Tsubame City, Niigata Prefecture, Japan. This project was jointly implemented by Nagaoka University of Technology and the Tsubame City Board of Education. The city has a total population of 75,931, comprising 58% working-age residents (15–64 years; *n* = 43,705) and 32% aged 65 or older (*n* = 24,312). Among them, 5,227 (6.9%) children aged 7–15 were enrolled in elementary and junior high schools. All households with school-aged children were invited to participate in the survey. While primary industries account for 4% of the population, secondary industries, particularly metal processing, represent a large portion (41%) of local employment, reflecting the city’s industrial character. No certified headache specialists or dedicated headache clinics are available in Tsubame City. The study site was selected based on geographic convenience and the feasibility of conducting a population-based study.

This survey was positioned as a preliminary study for a future complete population-based survey. Utilizing schools as distribution channels allowed efficient access to the working-age population through parents. After receiving approval from the Board of Education, survey invitations were disseminated through schools using both printed handouts and online communication tools. Since 2021, students have been using loaned tablet devices for online education during school closures triggered by the coronavirus disease-19 pandemic. Parents were requested to complete the questionnaire via Google Forms using either the provided tablets or their own smartphones. Only one parent per household was asked to respond, without specification of whether the father or mother should participate. All valid responses were defined as complete, with no missing data.

### Headache assessment and clinical characteristics

As initial exogenous variables in the SEM (also used as confounders in the multivariable regression model), we used age, biological sex, and headache characteristics (excluding diagnostic classification): We first collected information on the respondent’s age, biological sex, and the presence of headache attacks within the past three months, excluding those related to infections such as the common cold or head injuries. For those who reported having headaches, we further assessed the headache characteristics, including duration of headache attacks, monthly headache days (MHD), and the presence of specific symptoms: unilateral pain, pulsating pain, moderate or severe pain, aggravation by routine physical activity, nausea or vomiting, photophobia, phonophobia, and osmophobia [26], according to the ICHD-3 [5]. In addition, we collected the number of monthly acute medication intake days (AMD) and whether prophylactic medication had been taken in the past three months. Acute medications were indicated in the questionnaire as over-the-counter and prescribed non-steroidal anti-inflammatory drugs, triptans, and lasmiditan. Prophylactic medications were indicated as lomerizine, propranolol, valproic acid, amitriptyline, and calcitonin gene-related peptide (CGRP)-related drugs, according to the Japanese Clinical Practice Guideline for Headache Disorders 2021 [27]. Gepants had not yet been approved in Japan as of 2024.

While we did not ask whether respondents had received a formal medical diagnosis, we classified them as having migraine or medication-overuse headache (MOH) based on their responses, aligned with ICHD-3 definitions. These classifications were inferred from questionnaire items rather than clinical diagnoses, and they were not used as initial exogenous variables. These questionnaire items were also used in our previous study [28, 29].

### Working productivity assessment

As the outcome, we assessed work productivity loss caused by headache disorders using the WPAI Questionnaire-General Health [9]. Its reliability was shown among migraine patients [10]. WPAI is composed of four subscales: The percentage of work time missed due to headache (%lost, absenteeism), the percentage of impairment while working due to headache (%lost, presenteeism), degree of activity impairment due to headache (%lost, activity impairment), and degree of OWPI due to headache (%lost, OWPI) during the past seven days. Higher scores indicate greater work and activity impairment [10–12]. Of the four subscales, OWPI (%lost) was used as the primary outcome in this study because it comprehensively captures both absenteeism and presenteeism as work productivity impairment. OWPI was calculated using the following formula:

$$\eqalign{{\rm{OWPI }}\left( {{\rm{\% lost}}} \right){\rm{ = }} & {\rm{Absenteeism }}\left( {{\rm{\% lost}}} \right){\rm{ }} \cr & {\rm{ + }}\left[ {{\rm{100\% - Absenteeism }}\left( {{\rm{\% lost}}} \right)} \right]{\rm{ }} \cr & {\rm{ \times Presenteeism }}\left( {{\rm{\% lost}}} \right){\rm{.}} \cr} $$

Participants who were not engaged in paid work, such as housewives, were instructed to enter zero working hours in the questionnaire. Those who reported zero working hours during the past seven days were excluded from this analysis.

### Burden assessment

As mediators between clinical characteristics and OWPI, we assessed headache-related burden using two validated instruments: MIBS-4 for interictal burden and HIT-6 for ictal burden [19]. MIBS-4 measures the interictal burden of migraine across four areas using a five-point Likert scale: difficulties at work or school, disruptions in family and social life, challenges with planning or commitments, and emotional and cognitive distress. MIBS-4 score ranges from 0 to 12 [25]. In contrast, HIT-6 evaluates the overall ictal burden of headache on quality of life across six areas using a five-point Likert scale, including pain severity, functional limitations, energy levels, and emotional distress. HIT-6 score ranges from 36 to 78 [24]. The higher the score on either scale, the greater the burden.

### Structural equation model (SEM)

**Fig. 1:**
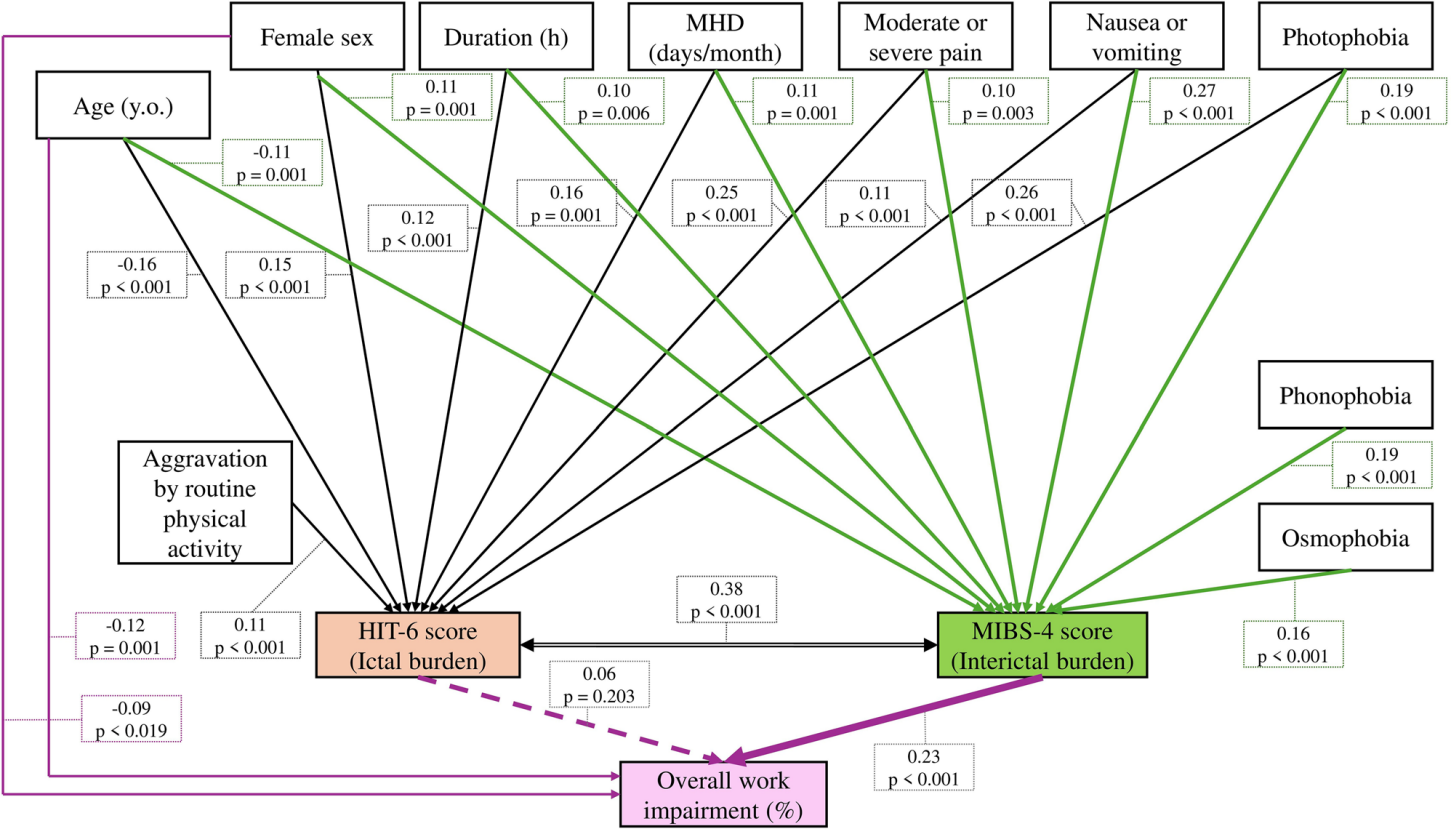
Structural Equation Model (SEM). This SEM illustrates relationships between headache symptoms, ictal burden (HIT-6), interictal anxiety (MIBS-4), and overall work impairment. Only statistically significant predictors (*p* < 0.05) from an initial full model were included. Maximum likelihood estimation with 1,000 bootstrap samples was used to account for non-normality. The numerical values described in the small box near the arrows show standardized coefficients (β). Solid arrows show direct paths, and the double-headed arrow represents standardized covariance

To investigate the association between MIBS-4 score and OWPI, and the indirect effect of clinical characteristics on OWPI through MIBS-4 score, we used SEM. SEM provides a robust framework for evaluating hypothesized causal relationships in cross-sectional data, particularly through mediation analysis [30]. Although SEM cannot establish true causality in cross-sectional designs [30], it allows for the examination of plausible paths based on theoretical models [31, 32]. In this SEM (Fig. 1), OWPI was treated as the final outcome, and OWPI was assumed to be associated with both MIBS-4 and HIT-6 scores. The initial exogenous variables (explanatory variables) for MIBS-4, HIT-6 scores, and OWPI were clinical characteristics, including age, biological sex, duration of headache attacks, MHD, AMD, presence of prophylactic medication, and the presence of specific symptoms (unilateral pain, pulsating pain, moderate or severe pain, aggravation by routine physical activity, nausea or vomiting, photophobia, phonophobia, and osmophobia). Here, we refer to the effect of clinical characteristics on OWPI that is not mediated through MIBS-4 or HIT-6 as the direct effect, and to the effect mediated through these mediators as the indirect effect. MIBS-4 and HIT-6 scores were modeled as correlated variables, allowing for covariance between them to reflect their potential bidirectional relationship.

To investigate the direct effects, the SEM was initially constructed with all candidate variables and all paths. Variables with non-significant direct paths (*p* ≥ 0.05) were subsequently removed [32]. However, the paths from MIBS-4 and HIT-6 scores to OWPI were retained in the final model, regardless of their statistical significance, to test the associations of MIBS-4 and HIT-6 scores on OWPI. Model estimation was performed using maximum likelihood estimation with 1,000 bootstrap replications to account for the non-normality of the outcome variables [33]. Model fit was evaluated using multiple fit indices: Comparative Fit Index (CFI), Tucker-Lewis Index (TLI), Root Mean Square Error of Approximation (RMSEA), and Standardized Root Mean Square Residual (SRMR). A CFI value of 0.95 or above, a TLI of 0.95 or above, an RMSEA of 0.06 or below, and an SRMR of 0.08 or below were considered indicators of an acceptable model fit, suggesting a relatively good fit between the hypothesized model and the observed data [34]. Second, we examined the significant indirect effects in the SEM. Indirect effects were estimated using the bias-corrected bootstrap method with 1,000 replications. Finally, we compared the associations between the clinical characteristics, two mediators (MIBS-4 and HIT-6), and OWPI by examining the statistical significance and standardized effect sizes (β) of their direct and indirect effects.

### Sensitivity analysis

To verify the robustness of the SEM results, we constructed three multiple linear regression models that decompose the structure of the SEM [35]. First, MIBS-4 score was used as the dependent variable, and all the initial exogenous variables were included as explanatory variables. Second, HIT-6 score was used as the dependent variable with the same set of explanatory variables. Finally, we constructed a multiple regression model using initial exogenous variables, MIBS-4, and HIT-6 scores as explanatory variables, with OWPI as the dependent variable. All explanatory variables were entered simultaneously using the forced-entry method. Because residuals deviated from normality as assessed by the Shapiro-Wilk test, bootstrapping (1,000 resamples) was applied to estimate confidence intervals (CIs) of the regression coefficients [36]. We compared the results of this sensitivity analysis with the SEM results.

Additionally, to examine potential sex effects given the predominance of female participants (92.9%), we repeated the development of SEM and multivariable regression model among female participants only. Because the number of male participants was small (*n* = 48), a separate male-only analysis was not feasible.

### Statistical analysis

Descriptive statistics were presented as medians and interquartile ranges (Q1–Q3) for variables that did not follow a normal distribution. Normality was evaluated using the Shapiro–Wilk test. Categorical variables were summarized as frequencies and percentages. No a priori power calculation was performed; instead, the sample size was determined by aiming to include as many residents as possible. Statistical significance was defined as a two-tailed *p* < 0.05. The results of the SEM and multivariable analyses were presented as unstandardized coefficients (B) with 95% CIs, and standardized coefficients (β) were calculated to facilitate comparison across variables. β was calculated by multiplying the unstandardized coefficient by the ratio of the standard deviation of the predictor to the outcome variable. SPSS Statistics version 30.0.0 (IBM Corp., Armonk, NY, USA), Python 3.9.0, Pandas 2.0–2, semopy 2.0, and Matplotlib 3.5.1 were used.

### Ethical aspects

This study was approved by the Ethics Committee of Nagaoka University of Technology (approval number: 2025-03-07). The anonymous survey collected no personally identifiable information. Participants were provided with an explanation of the study’s purpose both in writing and on the online survey form. They proceeded only if they gave consent. Those who did not wish to participate could submit a blank form. Participation was entirely voluntary. All procedures followed the Declaration of Helsinki and STROBE guidelines. On the first screen of the Google Form, participants were informed that their anonymous responses would be used for research and were asked to give consent before beginning the survey. Some of the data in this study were previously used in another study evaluating different outcomes [29], and some respondents may overlap across studies.

## Results

### General Characteristics

Out of the 5,227 households contacted, a total of 1,127 (21.6%) responses were obtained. Among these, 10 individuals explicitly declined participation, and 438 respondents reported no history of headache. Among the 679 respondents who experienced headaches, one did not complete the questionnaire and was excluded. No participants entered zero working hours during the past seven days; therefore, no respondents were excluded from the WPAI analysis. Consequently, 678 responses from parents with headaches (representing 13.0% of all invited households) were included in the final analysis.

**Fig. 2:**
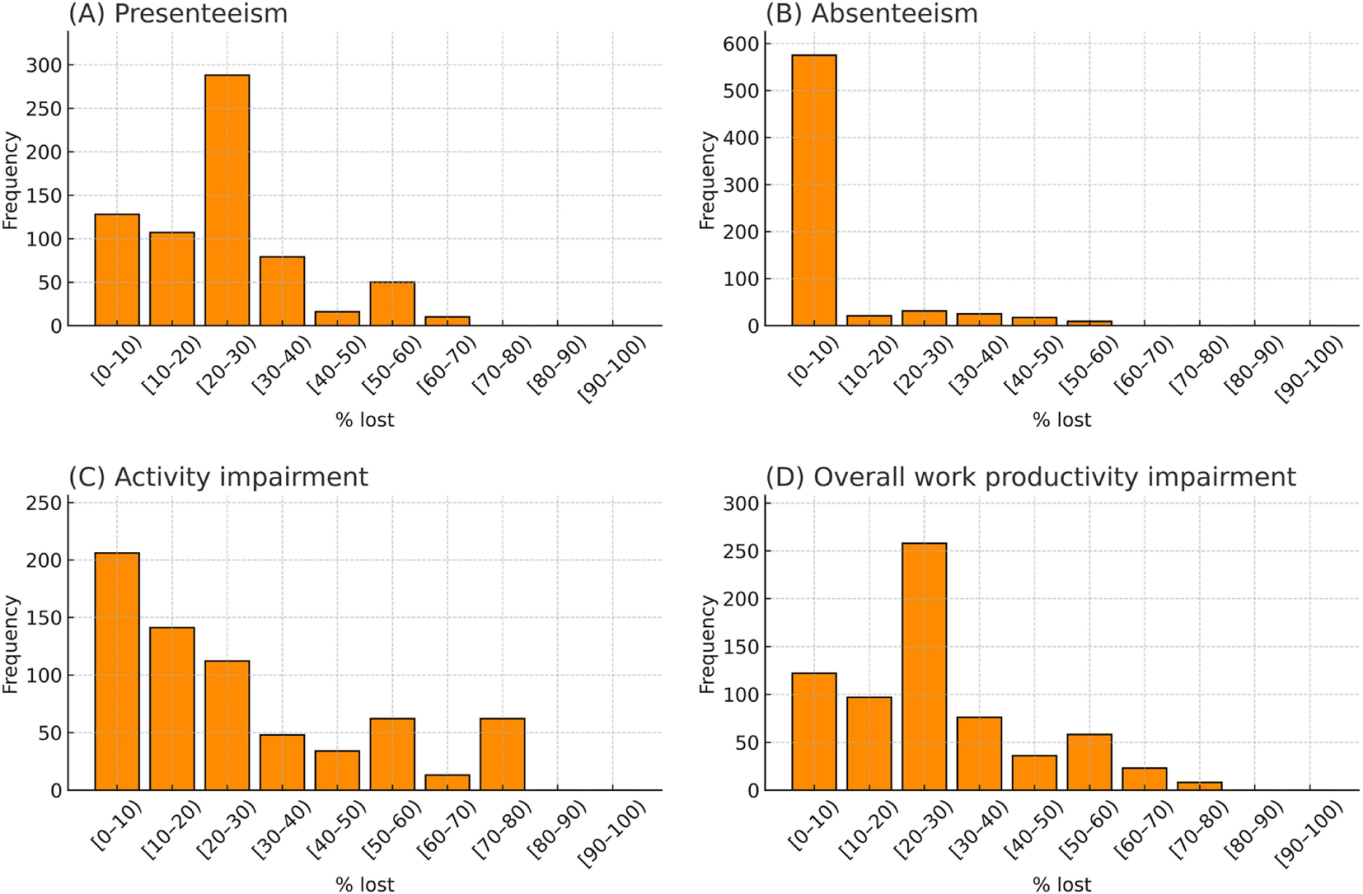
Distribution of Work Productivity and Activity Impairment (WPAI). Histograms illustrate the distributions of WPAI subscales OWPI among participants with headache disorders (*n* = 678). (**A**) presenteeism, (**B**) absenteeism, (**C**) activity impairment, and (**D)** overall work productivity impairment (OWPI). Each bar represents a 10% interval, with the lower bound included and the upper bound excluded; for example, [0–10%) includes values ≥ 0% and < 10%

The median (Q1–Q3) age was 43 (39–47) years, and 92.9% (630/678) were female. They reported median MHD: 3 (2–5) days, and AMD: 2 (1–5) days. Of the 678 respondents, 81.3% (551) used acute medications, and 7.8% (53) used prophylactic medications. Based on the response in the survey and classification according to ICHD-3, migraine comprised 21.2% (144), MOH 4.1% (28), both of them 1.2% (8), and others (73.5%; 498) did not fit the criteria for either migraine or MOH (Table 1). None of the patients used lasmiditan or CGRP-related drugs. Regarding interictal and ictal burden related to headache, the median MIBS-4 score was 4 (2–6), and the median HIT-6 score was 58 (53–64) (Table 2). Regarding WPAI, median presenteeism (%lost) was 20.0% (10.0%–20.0%) (Fig. 2A), median absenteeism was 0% (0%–4.9%) (Fig. 2B), median activity impairment was 10.0% (0%–40.0%) (Fig. 2C), and median OWPI was 20.0% (10.0%–30.0%) (Fig. 2D).

**Table 1:** Clinical characteristics (*n* = 678)

Variables	Median or number	IQR (Q1 – Q3) or %
Age (years)	43	39–47
Female sex	630	92.9%
**Headache characteristics**		
Duration of headache (h)	8	4–24
MHD (days/month)	3	2–5
Unilateral pain	349	51.5%
Pulsating pain	334	49.3%
Moderate or severe pain	138	20.4%
Aggravation by routine physical activity	123	18.1%
Nausea or vomiting	435	64.2%
Photophobia	162	23.9%
Phonophobia	289	42.6%
Osmophobia	107	15.8%
**Treatment**		
AMD (days/month)	2	1–5
Use of acute medication	551	81.3%
Use of prophylactic medication	53	7.8%
**Diagnosis**		
Migraine (+) and MOH (-)	144	21.2%
Migraine (-) and MOH (+)	28	4.1%
Migraine (+) and MOH (+)	8	1.2%
Migraine (-) and MOH (-)	498	73.5%

Abbreviations: AMD; acute medication intake days, IQR; interquartile range, MHD; monthly headache days, MOH; medication-overuse headache, NSAIDs; non-steroidal anti-inflammatory drugs, OTC; over-the-counter, Q1; first quartile, Q3; third quartile. There are no missing values

**Table 2:** Headache burden and work productivity and activity impairment (WPAI) (*n* = 678)

Variables	Median	IQR (Q1 – Q3)
**Burden**		
MIBS-4 score (sum)	4	2–6
HIT-6 score (sum)	58	53–64
**WPAI**		
Presenteeism (%lost)	20.0%	10.0%–20.0%
Absenteeism (%lost)	0%	0%–4.9%
Activity impairment (%lost)	10.0%	0%–40.0%
OWPI (%lost)	20.0%	10.0%–30.0%

HIT-6: Headache Impact Test-6, MIBS-4: Migraine Interictal Burden Scale-4, OWPI: overall work productivity impairment, WPAI: Work Productivity and Activity Impairment. There are no missing values

### SEM results

Figure 1 shows the final SEM results. The association between MIBS-4 and OWPI was confirmed, showing that greater MIBS-4 score was significantly associated with a greater OWPI (B = 3.12, [1.83–4.41]). However, the effect of HIT-6 on OWPI was not confirmed (B = 0.30, [-0.17–0.78]). Both MIBS-4 and HIT-6 scores were significantly correlated (unstandardized covariance = 8.10, [6.85–9.35], correlation coefficient; *r* = 0.38, *p* < 0.001), suggesting that interictal and ictal burdens co-occur and may associate with each other. The other significant paths are shown in Supplementary Table 1. Younger age and male sex had direct effects on OWPI, but other direct effects from clinical characteristics to OWPI were not confirmed.

In addition, the indirect effects of clinical characteristics on OWPI mediated by the MIBS-4 score were confirmed: younger age (B = -0.20, [-0.34–-0.09]), female sex (B = 3.65, [1.47–6.18]), longer duration of headache (B = 0.05, [0.01–0.08]), greater MHD (B = 0.20, [0.05–0.38]), presence of moderate/severe pain (B = 2.27, [0.59–4.15]), nausea/vomiting (B = 4.87, [2.94–7.16]), photophobia (B = 3.91, [1.95–6.29]), phonophobia (B = 3.36, [1.77–5.32]), and osmophobia (B = 3.78, [1.94–6.03]) were indirectly associated with OWPI mediated by MIBS-4 score. However, the indirect effects via HIT-6 were not confirmed (Supplementary Table 2). The SEM demonstrated a good fit: CFI = 0.962, TLI = 0.950, RMSEA = 0.047, and SRMR = 0.053, all of which indicate an acceptable fit to the data. These results suggest that MIBS-4 score as an interictal psychological burden plays a more substantial role in OWPI than HIT-6 score as an ictal headache burden. 

### Sensitivity analysis

In addition to the SEM analysis, we conducted sensitivity analyses using three multiple linear regression models that decomposed the SEM structure. The regression models consistently showed that multiple clinical characteristics were associated with increased MIBS-4 (Supplementary Table 3) and HIT-6 (Supplementary Table 4) scores. Furthermore, only MIBS-4 score but not HIT-6 score remained significantly associated with OWPI (Supplementary Table 5). These results were consistent with the SEM findings shown in Fig. 1 and Supplementary Table 1. This reinforced the robustness of our findings that MIBS-4 score had a strong association with OWPI, whereas HIT-6 score did not.

As a sensitivity analysis for female participants, we repeated the development of SEM and multivariable regression models among only female participants (*n* = 630). The general findings were reproduced; the association between MIBS-4 score and OWPI remained significant, whereas HIT-6 score was not. This result was consistent with the main analysis, suggesting that the predominance of female participants did not affect the conclusion. (Supplementary Tables 6 and 7 for SEM and Supplementary Tables 8–10 for multiple regression models).

## Discussion

This is the first study to investigate the relationship between MIBS-4 score and OWPI, in contrast to HIT-6 score. In our cohort, although MIBS-4 and HIT-6 scores correlated with each other, OWPI was associated only with MIBS-4 score (interictal burden), not HIT-6 score (ictal burden). Additionally, clinical characteristics were indirectly associated with OWPI mediated only via MIBS-4 score, but not HIT-6 score. Our findings indicate that interictal anticipatory anxiety and psychological distress between headache attacks may have a more profound impact on productivity loss than the burden of the attacks themselves. However, headache types were inferred from symptom-based questions and not confirmed by medical professionals, which may limit the external validity and generalizability of these findings.

### Headache disorders and WPAI

Our study revealed the distribution of WPAI subscales; median presenteeism (%lost) was 20.0%, median absenteeism was 0%, median activity impairment was 10.0%, and median OWPI was 20.0% (Fig. 2A and D). These distributions and their trends are largely consistent with previous reports on WPAI among individuals with headache disorders.

Matsumori et al. investigated the OVERCOME-Japan dataset, which is from a cross-sectional, observational, population-based web survey of 14,033 Japanese individuals with migraine. They demonstrated that absenteeism percentage was low as around 5%, while presenteeism, activity impairment, and OWPI percentages were around 40% [37]. Michael et al. used the data of 2,892 individuals with migraine from the National Health and Wellness Survey in European countries. They showed that absenteeism percentage was around 10%, while presenteeism, activity impairment, and OWPI percentages were around 40% [38]. Katsarava et al. used the dataset of the Chronic Migraine Epidemiology and Outcomes-International (CaMEO-I) study, a cross-sectional web-based survey performed across six countries in 2021, with 14,492 respondents with migraine. They demonstrated that the absenteeism percentage was 7%, while that of presenteeism was 41% [39].

In our results, the absenteeism percentage was low, whereas the percentages for presenteeism, activity impairment, and OWPI were high. This trend is consistent with these previous reports on migraine. The generally lower absolute percentages observed in our study may be attributed to the inclusion of various headache disorders, not only migraine, because migraine typically imposes a greater burden than other headache disorders like tension-type headache [40]. Additionally, the lower absenteeism observed in our study may reflect the cultural tendency in Japan to avoid taking leave from work, even when unwell [37, 41]. Taken together, the WPAI percentages observed in our study are reasonable and valid.

### Clinical characteristics associated with WPAI

In our study, OWPI was associated with MIBS-4 score, not HIT-6 score (Fig. 1 and Supplementary Table 1). Several clinical characteristics were indirectly associated with OWPI mediated only via MIBS-4 score, but not HIT-6 score. Additionally, younger age and male sex were directly associated with OWPI (Supplementary Table 2). To date, only two papers have conducted multivariate analyses to investigate the headache characteristics that affect WPAI [13, 15], and we herein compare their findings.

Naik et al. investigated 441 individuals in Canada with migraine. They found that a higher Migraine Disability Assessment (MIDAS) score was independently associated with higher WPAI after adjusting for sex, age, and other socioeconomic factors [14]. MIDAS, similar to HIT-6, is an indicator that evaluates the ictal burden of headache disorders [42]. However, their study did not evaluate interictal burden using the MIBS-4. In contrast, our study is novel in incorporating both ictal and interictal burdens. Therefore, their findings may reflect only the ictal aspect of headache burden, or they may be a result of the correlation between MIBS-4 and HIT-6 scores shown in our SEM. Rather, the results of their study and ours are not contradictory, but instead provide complementary perspectives. While their research highlights the impact of headache attacks themselves (ictal burden), our study emphasizes the importance of the burden experienced between attacks (interictal burden). Together, these studies provide a more comprehensive understanding of how headache disorders affect work productivity through the two aspects of ictal and interictal burdens. Further research focusing on both the interictal and ictal burden may be necessary in the future.

Kim et al. investigated 362 individuals with migraine or probable migraine in Korea. They found that male sex, higher frequency of acute medication use, and longer duration of migraine attack are associated with severe absenteeism after adjusting for age, sex, and other headache characteristics [16]. Consistent with their findings, our model identified a direct negative association between male sex and OWPI. One plausible explanation is that men may be less likely to seek medical care or adopt coping strategies [43], leading to under-treatment, migraine progression, and higher functional impairment. The association between longer headache duration and reduced productivity observed in our study is consistent with their findings. Longer attacks may increase anticipatory anxiety during headache-free periods, increasing interictal burden and further impairing work productivity. However, the association between frequent acute medication use and impaired work productivity in their study was not replicated in ours. This discrepancy may be due to two competing explanations: On one hand, individuals who frequently use acute medications might benefit from effective symptom relief, potentially leading to improved work productivity. On the other hand, MOH [44] or increased anticipatory anxiety related to acute medication use [45] could reduce productivity. Further studies are needed to elucidate which clinical characteristics may be associated with WPAI, with a focus on factors modified by treatments.

### Treatment strategies to improve WPAI

Our findings suggest some targets for improving WPAI among individuals with headache disorders. Treatment strategies can be categorized into three main approaches: (1) treatments that alleviate the MIBS-4 score and indirectly improve the WPAI, (2) treatments that directly improve the WPAI, and (3) conventional prophylactic treatments that mitigate headache frequency and severity.

#### (1) Treatments indirectly improving WPAI by alleviating the MIBS-4 score

Considering the association between MIBS-4 score and OWPI in our model, treatments targeting and alleviating MIBS-4 score are critical for improving OWPI. In CONQUER, galcanezumab significantly improved MIBS-4 score during the last 4 weeks of the 12-week period, with changes from baseline of -1.9 compared to -0.8 in the placebo group [46]. In GIANT, atogepant significantly improved MIBS-4 score in the same period, with changes from the baseline of -4.8 in a single-arm study [47]. Given that MIBS-4 is a newly developed instrument [25], there is limited evidence on interventions that directly improve MIBS-4 score.

Several clinical and psychosocial factors are known to increase MIBS-4 score. Higher HIT-6 score [48], severe pain [49] migraine with aura (not migraine without aura) [48, 49], weather changes and anxiety in causes of headache attacks [48], daytime sleepiness [48], low socioeconomic status [49], and low quality of life score [49] are associated with higher MIBS-4 score. Therefore, targeting these factors associated with MIBS-4 score may help improve WPAI. Various prophylactic medications, including conventional and recent CGRP-related drugs, improve HIT-6 score [50]. Some Japanese herbal kampo medicine improves weather-related headache attacks [51–53]. CGRP-related monoclonal antibodies improve anxiety [54]. Behavioral therapy, including cognitive behavioral therapy (CBT), biofeedback, relaxation training, mindfulness-based therapies, and/or education, improved anxiety and headache severity [55]. Psychological sleep interventions, such as sleep restriction, stimulus control, sleep hygiene, relaxation training, and cognitive restructuring, improve headache frequency and daytime sleepiness [56]. Further investigation is needed to confirm these treatments improve WPAI through alleviating MIBS-4 score.

#### (2) Treatments directly improving WPAI

Some drugs have been shown directly to improve WPAI in their clinical trials. In the DELIVER trial, in adults with migraine and prior prophylactic treatment failure, intravenous eptinezumab 100 mg and 300 mg administered every 12 weeks significantly improved approximately 20% of WPAI subscales over 4 weeks [57]. In the LIBERTY trial, the significant change from baseline in WPAI relative to placebo varied from -2.6% to -13.1% after treatment with erenumab 140 mg, reflecting improvements in 3 of the 4 WPAI subscales [58]. In the CONQUER trial, OWPI improved by 14.3% with galcanezumab compared to 3.5% with placebo after 12 weeks [59]. In the FOCUS trial for patients with episodic or chronic migraine, fremanezumab administered quarterly or monthly improved all four WPAI subscales, with significant changes from baseline ranging from -4.7% to -20.0% during the last four weeks of the 12-week double-blind treatment period compared to placebo except for absenteeism with quarterly fremanezumab [60]. Lastly, in the HALO-CM trial for patients with chronic migraine, quarterly or monthly fremanezumab significantly improved all four WPAI subscales during the last four weeks of the 12-week double-blind treatment period, with changes from baseline ranging from -12.9% to -16.6% [61]. In addition to CGRP-related drugs, a 36-week prospective real-world study found that onabotulinumtoxinA and topiramate both improved OWPI by around 1% [62]. In a separate 24-week randomized clinical trial, topiramate reduced mean monthly lost work hours from 14.6 to 5.1 h, representing a 65% relative reduction in time lost due to migraine [63]. Additionally, education on headache disorders through e-learning (45 min) and online consultations with headache specialists have improved work productivity. For employees with headaches who received virtual consultations, the number of headache days with at least 50% productivity loss decreased from 16.7 days to 13.3 days per three months. This study indicates that non-drug treatments are also effective [64].

#### (3) Conventional prophylactic treatments

Conventional prophylactic medications and CBT have been shown to reduce MHD, duration of attack, and pain intensity [65, 66], which may indirectly improve OWPI possibly through improving MIBS-4 scores. However, evidence supporting the direct impact of these treatments on WPAI and MIBS-4remains limited. Future research should investigate whether these treatments can directly improve WPAI or indirectly through MIBS-4.

### Limitations

First, the survey was conducted among parents of school-aged children in Japan, and thus its generalizability is limited. The low response rate (21.6%) may have led to selection bias and raised the possibility of non-response bias. Besides, the study population primarily consisted of mothers (92.9%) and excluded important populations such as adults without children, younger and older adults/parents, as well as fathers with headache disorders. Especially, the predominance of female respondents may have associated the psychological burden findings due to existing gender-based disparities in employment and caregiving roles in Japan. In Japan, significant sex-based disparities persist in employment conditions. As of 2023, approximately 54% of employed women were in non-regular positions (part-time, contract, or temporary), compared to 22% of men. Women are also underrepresented in full-time employment and leadership roles; only about 15% of managerial positions are held by women. Although the female labor force participation rate has increased to around 74%, surpassing the OECD average, women still face disadvantages in job stability, wages, and career development [67]. This working environment among Japanese females is related to psychological burden [68], but we did not investigate such socioeconomic factors. To address these limitations, future studies should consider population-based surveys covering entire communities or internet-based surveys designed to reflect the national demographic structure. Such approaches would enable the inclusion of underrepresented groups, including fathers, childless individuals, and people across various age groups.

Second, this study utilized self-reported data collected through an online survey. Although headache types were inferred based on symptom-based questions consistent with ICHD-3 criteria, participants did not receive formal diagnoses of migraine or MOH by medical professionals. Therefore, the classification of “headache” in our analysis encompasses a broad spectrum, including migraine, tension-type headache, and potentially other forms, each of which may differently influence WPAI. Additionally, self-reporting introduces the possibility of recall bias. Diagnostic software comparable to physician’s diagnostic performance using artificial intelligence may solve this problem in the research using questionnaire [69, 70]. Furthermore, the SEM and cross-sectional nature of the study also limit our ability to conclude true causality. We used SEM, which implies the directions and associations among factors. However, the cross-sectional nature of this study cannot definitely remove the possibility of reverse causality (e.g., poor work productivity contributing to higher interictal burden). To overcome these limitations, future studies should adopt a longitudinal design that ideally incorporates prospective headache diaries to reduce recall bias and enable the observation of temporal relationships and causality between headache disorders and WPAI. Interventional studies targeting interictal burden and assessing WPAI are also essential. Besides, psychological and basic studies to explore the mechanisms underlying the interictal burden and WPAI. Furthermore, clinical confirmation of headache diagnoses by physicians will also be essential for enhancing diagnostic accuracy in subsequent research.

In addition, we did not conduct an a priori power analysis, because the expected effect sizes of individual SEM paths were difficult to anticipate in advance. Although our sample size of 678 respondents is generally considered adequate for the number of estimated parameters, this remains a limitation. Future studies should incorporate prospective sample size and power calculations to further strengthen statistical rigor.

Finally, we utilized HIT-6, MIBS-4, and OWPI to assess headache burden and work productivity. However, other validated scales also evaluate them, such as MIDAS and Migraine-Specific Quality of Life Questionnaire (MSQ) [71]. The selection and structure of these measurement tools may have influenced the interpretation of our SEM results.

## Conclusions

Although MIBS-4 and HIT-6 scores were moderately correlated, OWPI was associated with MIBS-4 score (interictal burden), not HIT-6 score (ictal burden). Additionally, clinical characteristics are indirectly associated with OWPI mediated via the MIBS-4 score, but not the HIT-6 score. Our findings suggest that interictal anticipatory anxiety and psychological distress between headache attacks may have a more profound impact on productivity loss than the burden of the attacks themselves. Addressing the interictal psychological burden, in addition to the ictal burden, may be essential for improving work productivity among individuals with headache disorders.

## Study highlights

1. The Migraine Interictal Burden Scale-4 (MIBS-4) measures interictal burden, while the Headache Impact Test-6 (HIT-6) measures ictal burden.
2. We investigated the relationship between Work Productivity and Activity Impairment (WPAI) and MIBS-4 score, in contrast to HIT-6 score.
3. Overall work productivity impairment (OWPI), as measured by WPAI, was associated with the MIBS-4 score, but not by the HIT-6 score.
4. Clinical characteristics are indirectly associated with OWPI mediated via the MIBS-4 score, but not the HIT-6 score.
5. Addressing the interictal burden may be essential for improving work productivity.

## Supplementary Information

Below is the link to the electronic supplementary material.

Supplementary Material 1

## Data Availability

The datasets generated and/or analyzed during the current study are available from the corresponding author upon reasonable request.

## Abbreviations

AMD: Monthly acute medication intake days
CaMEO-I: Chronic Migraine Epidemiology and Outcomes-International
CBT: Cognitive behavioral therapy
CGRP-mAbs: Monoclonal antibodies targeting calcitonin gene-related peptide
CI: Confidence interval
HIT-6: Headache Impact Test-6
ICHD-3: International Classification of Headache Disorders, 3rd edition
MHD: Monthly headache days
MIBS-4: Migraine Interictal Burden Scale-4
MIDAS: Migraine Disability Assessment
MOH: Medication-overuse headache
OECD: Organisation for Economic Co-operation and Development
OR: Odds ratio
OWPI: Overall work productivity impairment
SEM: Structural equation model
SRMR: Standardized root mean square residual
RMSEA: Root mean square error of approximation
TLI: Tucker–Lewis Index
CFI: Comparative Fit Index
WPAI: Work Productivity and Activity Impairment
