# Supplementary material for: Interictal burden, not ictal burden, drives work productivity impairment in headache disorders: insights from a Japanese cross-sectional study
Source: J Headache Pain. 2026 Feb 3;27(1):63. doi: 10.1186/s10194-026-02284-4 (PMC12955254; doi:10.1186/s10194-026-02284-4)
Supplement: Supplementary file 1 — Supplementary Material 1 [file 10194_2026_2284_MOESM1_ESM.docx]

**Supplementary Table 1 Parameter Estimates of the Paths in the Final Structural Equation Model Corresponding to Figure 1**

| Variables | Unstandardized coefficient (B) | 95% confidence interval for B | Standardized coefficient (β) | 95% confidence interval for β | p values |
| --- | --- | --- | --- | --- | --- |
| MIBS-4 score and clinical characteristics |  |  |  |  |  |
| MIBS-4 ~ Age | -0.06 | -0.10 – -0.03 | -0.11 | -0.18 – -0.06 | 0.001* |
| MIBS-4 ~ Female sex | 1.17 | 0.45 – 1.89 | 0.11 | 0.04 – 0.18 | 0.001* |
| MIBS-4 ~ Duration of headache | 0.01 | 0.00 – 0.02 | 0.10 | 0.00 – 0.20 | 0.006* |
| MIBS-4 ~ Monthly headache days | 0.06 | 0.02 – 0.10 | 0.11 | 0.04 – 0.18 | 0.001* |
| MIBS-4 ~ Moderate or severe pain | 0.72 | 0.25 – 1.19 | 0.10 | 0.04 – 0.17 | 0.003* |
| MIBS-4 ~ Nausea or vomiting | 1.57 | 1.14 – 2.00 | 0.27 | 0.20 – 0.34 | < 0.001* |
| MIBS-4 ~ Photophobia | 1.25 | 0.82 – 1.69 | 0.19 | 0.13 – 0.26 | < 0.001* |
| MIBS-4 ~ Phonophobia | 1.08 | 0.70 – 1.46 | 0.19 | 0.12 – 0.26 | < 0.001* |
| MIBS-4 ~ Osmophobia | 1.20 | 0.76 – 1.63 | 0.16 | 0.10 – 0.22 | < 0.001* |
| HIT-6 score and clinical characteristics |  |  |  |  |  |
| HIT6 ~ Age | -0.25 | -0.34 – -0.15 | -0.16 | -0.22 – -0.10 | < 0.001* |
| HIT6 ~ Female sex | 4.53 | 2.66 – 6.40 | 0.15 | 0.09 – 0.21 | < 0.001* |
| HIT6 ~ Duration of headache | 0.05 | 0.02 – 0.07 | 0.12 | 0.05 – 0.17 | < 0.001* |
| HIT6 ~ Monthly headache days | 0.25 | 0.15 – 0.35 | 0.16 | 0.10 – 0.22 | 0.001* |
| HIT6 ~ Moderate or severe pain | 4.73 | 3.49 – 5.97 | 0.25 | 0.18 – 0.32 | < 0.001* |
| HIT6 ~ Aggravation by routine physical activity | 2.16 | 1.06 – 3.26 | 0.11 | 0.05 – 0.17 | < 0.001* |
| HIT6 ~ Nausea or vomiting | 1.82 | 0.84 – 2.81 | 0.11 | 0.05 – 0.17 | < 0.001* |
| HIT6 ~ Photophobia | 4.76 | 3.63 – 5.89 | 0.26 | 0.20 – 0.32 | < 0.001* |
| Burdens, clinical characteristics, and final output |  |  |  |  |  |
| OPWI ~ Age | -0.89 | -1.44 – -0.35 | -0.12 | -0.19 – -0.05 | 0.001* |
| OPWI ~ Female sex | -12.78 | -23.47 – -2.10 | -0.09 | -0.17 – -0.02 | 0.019* |
| OPWI ~ MIBS-4 | 3.12 | 1.83 – 4.41 | 0.23 | 0.14 – 0.33 | < 0.001* |
| OPWI ~ HIT-6 | 0.30 | -0.17 – 0.78 | 0.06 | -0.03 – 0.16 | 0.203 |
| MIBS-4 ~~ HIT-6 (Covariance) | 8.11 | 6.85 – 9.35 | 0.38 (r) | 0.32 – 0.43 | < 0.001* |

This table presents the unstandardized (B) and standardized (β) path coefficients estimated in the final structural equation model (SEM) shown in **Figure 1**. Only statistically significant predictors (p < 0.05) were retained in the final model, except for the non-significant path from Headache Impact Test-6 (HIT-6) score to overall work productivity impairment (OWPI). β was calculated by multiplying the unstandardized coefficient by the ratio of the standard deviation of the predictor to the outcome variable. The covariance between Migraine Interictal Burden Scale-4 (MIBS-4) and HIT-6 scores is also shown, along with its standardized covariance (Pearson's r). Confidence intervals were estimated using bootstrapping with 1,000 resamples. Asterisks indicate statistical significance (p < 0.05). There are no missing values.

**Supplementary Table 2 Parameter Estimates of the Indirect Effects in the Final Structural Equation Model Corresponding to Figure 1**

| Variables | Unstandardized coefficient (B) | 95% confidence interval for B | Standardized coefficient (β) | 95% confidence interval for β | p values |
| --- | --- | --- | --- | --- | --- |
| MIBS-4-score-mediated effects |  |  |  |  |  |
| OWPI - MIBS4 - Age | -0.20 | -0.34 – -0.09 | -0.027 | -0.05 – -0.01 | < 0.001* |
| OWPI - MIBS4 - Female sex | 3.65 | 1.47 – 6.18 | 0.025 | 0.01 – 0.04 | 0.002* |
| OWPI - MIBS4 - Duration of headache | 0.05 | 0.01 – 0.08 | 0.023 | 0.01 – 0.04 | 0.010* |
| OWPI - MIBS4 - Monthly headache days | 0.20 | 0.05 – 0.38 | 0.026 | 0.01 – 0.05 | 0.002* |
| OWPI - MIBS4 - Moderate or severe pain | 2.27 | 0.59 – 4.15 | 0.024 | 0.01 – 0.04 | 0.010* |
| OWPI - MIBS4 - Nausea or vomiting | 4.87 | 2.94 – 7.16 | 0.062 | 0.04 – 0.09 | < 0.001* |
| OWPI - MIBS4 - Photophobia | 3.91 | 1.95 – 6.29 | 0.044 | 0.02 – 0.07 | < 0.001* |
| OWPI - MIBS4 - Phonophobia | 3.36 | 1.77 – 5.32 | 0.044 | 0.02 – 0.07 | < 0.001* |
| OWPI - MIBS4 - Osmophobia | 3.78 | 1.94 – 6.03 | 0.036 | 0.02 – 0.06 | < 0.001* |
| HIT-6-score-mediated effects |  |  |  |  |  |
| OWPI - HIT6 - Age | -0.08 | -0.21 – 0.03 | -0.01 | -0.03 – 0.00 | 0.175 |
| OWPI - HIT6 - Female sex | 1.44 | -0.48 – 3.83 | 0.009 | 0.00 – 0.02 | 0.184 |
| OWPI - HIT6 - Duration of headache | 0.01 | -0.01 – 0.04 | 0.007 | -0.01 – 0.03 | 0.160 |
| OWPI - HIT6 - Monthly headache days | 0.08 | -0.03 – 0.22 | 0.010 | 0.00 – 0.03 | 0.138 |
| OWPI - HIT6 - Moderate or severe pain | 1.52 | -0.54 – 3.70 | 0.015 | -0.01 – 0.04 | 0.152 |
| OWPI - HIT6 - Aggravation by routine physical activity | 0.65 | -0.28 – 1.80 | 0.007 | 0.00 – 0.02 | 0.176 |
| OWPI - HIT6 - Nausea or vomiting | 0.56 | -0.21 – 1.52 | 0.007 | 0.00 – 0.02 | 0.191 |
| OWPI - HIT6 - Photophobia | 1.43 | -0.64 – 3.73 | 0.016 | -0.01 – 0.04 | 0.179 |

This table presents the unstandardized (B) and standardized (β) indirect effect coefficients estimated in the final structural equation model (SEM) shown in **Figure 1**. Abbreviations; HIT-6:

Headache Impact Test-6, MIBS-4: Migraine Interictal Burden Scale-4, OWPI: overall work productivity impairment. There are no missing values.

**Supplementary Table 3 Multivariable Linear Regression Analysis of Headache Characteristics Associated with Migraine Interictal Burden Scale (MIBS-4) Score**

| Variables | Unstandardized coefficient (B) | 95% confidence interval for B | Standardized coefficient (β) | 95% confidence interval for β | p values |
| --- | --- | --- | --- | --- | --- |
| Age (years) | -0.06 | -0.09 – -0.03 | -0.11 | -0.17 – -0.06 | 0.002* |
| Female sex | 1.09 | 0.44 – 1.81 | 0.10 | 0.04 – 0.17 | < 0.001* |
| Duration of headache (h) | 0.01 | 0.00 – 0.02 | 0.09 | 0.00 – 0.18 | 0.040* |
| MHD (days/month) | 0.04 | -0.03 – 0.10 | 0.06 | -0.05 – 0.15 | 0.255 |
| Unilateral pain | -0.12 | -0.48 – 0.28 | -0.02 | -0.08 – 0.05 | 0.552 |
| Pulsating pain | -0.23 | -0.61 – 0.16 | -0.04 | -0.11 – 0.03 | 0.245 |
| Moderate or severe pain | 0.69 | 0.10 – 1.28 | 0.10 | 0.01 – 0.19 | 0.024* |
| Aggravation by routine physical activity | -0.14 | -0.66 – 0.37 | -0.02 | -0.09 – 0.05 | 0.617 |
| Nausea or vomiting | 1.57 | 1.15 – 1.99 | 0.27 | 0.20 – 0.34 | < 0.001* |
| Photophobia | 1.22 | 0.78 – 1.68 | 0.18 | 0.12 – 0.25 | < 0.001* |
| Phonophobia | 1.17 | 0.74 – 1.60 | 0.21 | 0.13 – 0.29 | < 0.001* |
| Osmophobia | 1.83 | 1.37 – 2.31 | 0.24 | 0.18 – 0.30 | < 0.001* |
| AMD (day/month) | 0.03 | -0.02 – 0.08 | 0.06 | -0.04 – 0.16 | 0.236 |
| Use of prophylactic medication | -0.11 | -0.92 – 0.70 | -0.01 | -0.08 – 0.06 | 0.779 |

This table presents the results of a multivariable linear regression analysis with 1,000 bootstrap samples examining which headache characteristics were associated with MIBS-4 score. All explanatory variables were entered simultaneously using the forced-entry method. P-values less than 0.05 were considered statistically significant and are marked with an asterisk (*). The linear regression model demonstrated acceptable explanatory power, with an adjusted R² of 0.308. The overall model was statistically significant (F(14, 663) = 22.486, p < 0.001). Bootstrapping confirmed the stability of the estimates (bias = -0.720, standard error = 0.072, 95% CI [1.176, 1.460]). MHD: monthly headache days; AMD: acute medication days. There are no missing values.

**Supplementary Table 4 Multivariable Linear Regression Analysis of Headache Characteristics Associated with Headache Impact Test-6 (HIT-6) Score**

| Variables | Unstandardized coefficient (B) | 95% confidence interval for B | Standardized coefficient (β) | 95% confidence interval for β | p values |
| --- | --- | --- | --- | --- | --- |
| Age (years) | -0.24 | -0.34 – -0.14 | -0.16 | -0.23 – -0.09 | < 0.001* |
| Female sex | 4.11 | 2.14 – 6.15 | 0.14 | 0.07 – 0.21 | < 0.001* |
| Duration of headache (h) | 0.04 | 0.02 – 0.07 | 0.11 | 0.06 – 0.19 | 0.005* |
| MHD (days/month) | 0.19 | 0.01 – 0.36 | 0.12 | 0.01 – 0.23 | 0.032* |
| Unilateral pain | -0.50 | -1.49 – 0.50 | -0.03 | -0.09 – 0.03 | 0.310 |
| Pulsating pain | -0.31 | -1.33 – 0.81 | -0.02 | -0.09 – 0.05 | 0.577 |
| Moderate or severe pain | 4.53 | 3.30 – 6.01 | 0.24 | 0.18 – 0.32 | < 0.001* |
| Aggravation by routine physical activity | 1.43 | 0.00 – 2.73 | 0.07 | 0.00 – 0.13 | 0.041* |
| Nausea or vomiting | 1.96 | 0.91 – 2.96 | 0.12 | 0.06 – 0.18 | < 0.001* |
| Photophobia | 4.51 | 3.45 – 5.63 | 0.25 | 0.19 – 0.31 | < 0.001* |
| Phonophobia | 0.47 | -0.64 – 1.47 | 0.03 | -0.04 – 0.09 | 0.397 |
| Osmophobia | 2.94 | 1.82 – 4.23 | 0.14 | 0.09 – 0.20 | < 0.001* |
| AMD (day/month) | 0.07 | -0.08 – 0.22 | 0.05 | -0.06 – 0.16 | 0.383 |
| Use of prophylactic medication | -0.69 | -2.39 – 1.05 | -0.02 | -0.07 – 0.03 | 0.444 |

This table presents the results of a multivariable linear regression analysis with 1,000 bootstrap samples examining which headache characteristics were associated with HIT-6 score. All explanatory variables were entered simultaneously using the forced-entry method. P-values less than 0.05 were considered statistically significant and are marked with an asterisk (*). The linear regression model demonstrated acceptable explanatory power, with an adjusted R² of 0.371. The overall model was statistically significant (F(14, 663) = 29.498, p < 0.001). Bootstrapping confirmed the stability of the estimates (bias = -0.671, standard error = 0.075, 95% CI [1.092, 1.386]). MHD: monthly headache days; AMD: acute medication days. There are no missing values.

**Supplementary Table 5 Multivariable Linear Regression Analysis of Headache Characteristics Associated with Overall Work Productivity Impairment (OWPI)**

| Variables | Unstandardized coefficient (B) | 95% confidence interval for B | Standardized coefficient (β) | 95% confidence interval for β | p values |
| --- | --- | --- | --- | --- | --- |
| Age (years) | -0.84 | -1.39 - -0.31 | -0.11 | -0.18 – -0.04 | 0.004* |
| Female sex | -8.20 | -21.68 - 5.74 | -0.06 | -0.16 – 0.04 | 0.148 |
| Duration of headache (h) | -0.08 | -0.23 - 0.08 | -0.04 | -0.12 – 0.04 | 0.305 |
| MHD (days/month) | -0.04 | -0.86 - 0.83 | -0.01 | -0.22 – 0.21 | 0.934 |
| Unilateral pain | 1.13 | -4.34 - 6.43 | 0.02 | -0.08 – 0.11 | 0.686 |
| Pulsating pain | 0.21 | -5.53 - 6.23 | 0.00 | 0.00 – 0.00 | 0.942 |
| Moderate or severe pain | -2.58 | -9.32 - 3.76 | -0.03 | -0.11 – 0.04 | 0.520 |
| Aggravation by routine physical activity | -1.04 | -7.07 - 5.17 | -0.01 | -0.07 – 0.05 | 0.806 |
| Nausea or vomiting | -5.78 | -12.34 - 0.56 | -0.07 | -0.15 – 0.01 | 0.121 |
| Photophobia | 7.11 | 0.79 - 13.59 | 0.08 | 0.01 – 0.15 | 0.050 |
| Phonophobia | -12.45 | -19.32 - -5.97 | -0.16 | -0.25 – -0.08 | < 0.001* |
| Osmophobia | 5.29 | -1.86 - 13.32 | 0.05 | -0.02 – 0.13 | 0.222 |
| AMD (day/month) | 0.41 | -0.52 - 1.28 | 0.06 | -0.08 – 0.19 | 0.324 |
| Use of prophylactic medication | 2.46 | -5.59 - 10.44 | 0.02 | -0.05 – 0.09 | 0.669 |
| MIBS-4 score (sum) | 3.41 | 2.08 - 4.58 | 0.26 | 0.16 – 0.35 | < 0.001* |
| HIT-6 score (sum) | 0.21 | -0.33 - 0.71 | 0.04 | -0.06 – 0.14 | 0.133 |

This table presents the results of a multivariable linear regression analysis with 1,000 bootstrap samples examining whether MIBS-4 and HIT-6 scores were associated with OWPI. All explanatory variables were entered simultaneously using the forced-entry method. P-values less than 0.05 were considered statistically significant and are marked with an asterisk (*). The linear regression model demonstrated acceptable explanatory power, with an adjusted R² of 0.081. The overall model was statistically significant (F(2, 675) = 30.788, p < 0.001). Bootstrapping confirmed the stability of the estimates (bias = -0.792, standard error = 0.068, 95% CI [1.174, 1.435]). HIT-6: Headache Impact Test-6; MIBS-4: Migraine Interictal Burden Scale. There are no missing values.

**Supplementary Table 6 Parameter Estimates of the Paths in the Final Structural Equation Model among Female Participants**

| Variables | Unstandardized coefficient (B) | 95% confidence interval for B | Standardized coefficient (β) | 95% confidence interval for β | p values |
| --- | --- | --- | --- | --- | --- |
| MIBS-4 score and clinical characteristics |  |  |  |  |  |
| MIBS-4 ~ Age | -0.07 | -0.11 – -0.03 | -0.16 | -0.22 – -0.10 | < 0.001* |
| MIBS-4 ~ Duration of headache | 0.01 | 0.002 – 0.02 | 0.11 | 0.05 – 0.20 | 0.024* |
| MIBS-4 ~ Moderate or severe pain | 0.74 | 0.22 – 1.26 | 0.24 | 0.17 – 0.31 | 0.005* |
| MIBS-4 ~ Nausea or vomiting | 1.69 | 1.21 – 2.17 | 0.12 | 0.05 – 0.19 | < 0.001* |
| MIBS-4 ~ Photophobia | 1.30 | 0.83 – 1.78 | 0.25 | 0.18 – 0.32 | < 0.001* |
| MIBS-4 ~ Phonophobia | 1.20 | 0.71 – 1.68 | 0.03 | 0.01 – 0.11 | < 0.001* |
| MIBS-4 ~ Osmophobia | 1.77 | 1.21 – 2.32 | 0.14 | 0.06 – 0.21 | < 0.001* |
| HIT-6 score and clinical characteristics |  |  |  |  |  |
| HIT6 ~ Age | -0.27 | -0.36 – -0.17 | -0.16 | -0.22 – -0.11 | < 0.001* |
| HIT6 ~ Duration of headache | 0.04 | 0.02 – 0.07 | 0.11 | 0.05 – 0.19 | 0.001* |
| HIT6 ~ Monthly headache days | 0.17 | 0.03 – 0.32 | 0.12 | 0.02 – 0.21 | 0.010* |
| HIT6 ~ Moderate or severe pain | 4.88 | 3.54 – 6.21 | 0.24 | 0.17 – 0.31 | < 0.001* |
| HIT6 ~ Aggravation by routine physical activity | 1.64 | 0.21 – 3.06 | 0.07 | 0.01 – 0.13 | 0.002* |
| HIT6 ~ Nausea or vomiting | 2.10 | 0.68 – 3.31 | 0.12 | 0.05 – 0.19 | < 0.001* |
| HIT6 ~ Photophobia | 4.49 | 3.28 – 5.69 | 0.25 | 0.18 – 0.32 | < 0.001* |
| HIT6 ~ Osmophobia | 2.62 | 1.20 – 4.04 | 0.14 | 0.06 – 0.22 | < 0.001* |
| Burdens, clinical characteristics, and final output |  |  |  |  |  |
| OPWI ~ Age | -0.01 | -0.01 – -0.002 | -0.10 | -0.17 – -0.02 | 0.011* |
| OPWI ~ Nausea or vomiting | -0.09 | -0.16 – -0.01 | -0.11 | -0.21 – -0.01 | 0.020* |
| OPWI ~ Phonophobia | -0.15 | -0.22 – -0.08 | -0.19 | -0.28 – -0.10 | < 0.001* |
| OPWI ~ MIBS-4 | 0.03 | 0.02 – 0.05 | 0.26 | 0.16 – 0.37 | < 0.001* |
| OPWI ~ HIT-6 | 0.003 | -0.002 – 0.01 | 0.06 | -0.05 – 0.17 | 0.278 |
| MIBS-4 ~~ HIT-6 (Covariance) | 7.76 | 6.50 – 9.02 | 0.55 (r) | 0.49 – 0.60 | < 0.001* |

This table presents the unstandardized (B) and standardized (β) path coefficients estimated in the final structural equation model (SEM) among only female participants. Only statistically significant predictors (p < 0.05) were retained in the final model, except for the non-significant path from Headache Impact Test-6 (HIT-6) score to overall work productivity impairment (OWPI). β was calculated by multiplying the unstandardized coefficient by the ratio of the standard deviation of the predictor to the outcome variable. The covariance between Migraine Interictal Burden Scale-4 (MIBS-4) and HIT-6 scores is also shown, along with its standardized covariance (Pearson's r). Confidence intervals were estimated using bootstrapping with 1,000 resamples. Asterisks indicate statistical significance (p < 0.05). There are no missing values.

**Supplementary Table 7 Parameter Estimates of the Indirect Effects in the Final Structural Equation Model among Female Participants**

| Variables | Unstandardized coefficient (B) | 95% confidence interval for B | Standardized coefficient (β) | 95% confidence interval for β | p values |
| --- | --- | --- | --- | --- | --- |
| MIBS-4-score-mediated effects |  |  |  |  |  |
| OWPI - MIBS4 - Age | -0.21 | -0.55 – -0.06 | -0.041 | -0.06 – -0.01 | 0.004* |
| OWPI - MIBS4 - Duration of headache | 0.03 | 0.004 – 0.10 | 0.032 | 0.01 – 0.05 | 0.040* |
| OWPI - MIBS4 - Moderate or severe pain | 2.22 | 0.44 – 6.3 | 0.029 | 0.01 – 0.05 | 0.015* |
| OWPI - MIBS4 - Nausea or vomiting | 5.07 | 2.42 – 10.85 | 0.062 | 0.04 – 0.09 | < 0.001* |
| OWPI - MIBS4 - Photophobia | 3.90 | 1.66 – 8.90 | 0.044 | 0.02 – 0.07 | < 0.001* |
| OWPI - MIBS4 - Phonophobia | 3.60 | 1.42 – 8.4 | 0.044 | 0.02 – 0.07 | < 0.001* |
| OWPI - MIBS4 - Osmophobia | 5.31 | 2.42 – 11.60 | 0.036 | 0.02 – 0.06 | < 0.001* |
| HIT-6-score-mediated effects |  |  |  |  |  |
| OWPI - HIT6 - Age | -0.08 | -0.36 – 0.07 | -0.010 | -0.04 – 0.00 | 0.288 |
| OWPI - HIT6 - Duration of headache | 0.01 | -0.01 – 0.07 | 0.012 | -0.01 – 0.03 | 0.303 |
| OWPI - HIT6 - Monthly headache days | 0.05 | -0.06 – 0.32 | 0.009 | -0.01 – 0.03 | 0.324 |
| OWPI - HIT6 - Moderate or severe pain | 1.46 | -1.24 – 6.21 | 0.014 | -0.01 – 0.04 | 0.283 |
| OWPI - HIT6 - Aggravation by routine physical activity | 0.49 | -0.61 – 3.06 | 0.004 | -0.01 – 0.02 | 0.328 |
| OWPI - HIT6 - Nausea or vomiting | 0.63 | -0.66 – 3.31 | 0.010 | -0.02 – 0.05 | 0.302 |
| OWPI - HIT6 - Photophobia | 1.34 | -1.14 – 5.69 | 0.010 | -0.01 – 0.03 | 0.283 |
| OWPI - HIT6 - Osmophobia | 0.78 | -0.81 – 4.04 | 0.006 | -0.01 – 0.02 | 0.298 |

This table presents the unstandardized (B) and standardized (β) path coefficients estimated in the final structural equation model (SEM) among only female participants. Abbreviations; HIT-6:

Headache Impact Test-6, MIBS-4: Migraine Interictal Burden Scale-4, OWPI: overall work productivity impairment. There are no missing values.

**Supplementary Table 8 Multivariable Linear Regression Analysis of Headache Characteristics Associated with Migraine Interictal Burden Scale (MIBS-4) Score among Female Participants**

| Variables | Unstandardized coefficient (B) | 95% confidence interval for B | Standardized coefficient (β) | 95% confidence interval for β | p values |
| --- | --- | --- | --- | --- | --- |
| Age (years) | -0.07 | -0.10 – -0.03 | -0.12 | -0.17 – -0.05 | < 0.001* |
| Duration of headache (h) | 0.01 | 0.00 – 0.02 | 0.08 | 0.00 – 0.16 | 0.025* |
| MHD (days/month) | 0.04 | -0.18 – 0.10 | 0.07 | -0.31 – 0.15 | 0.185 |
| Unilateral pain | -0.17 | -0.40 – 0.36 | -0.01 | -0.02 – 0.02 | 0.929 |
| Pulsating pain | -0.20 | -0.61 – 0.19 | -0.04 | -0.12 – 0.04 | 0.313 |
| Moderate or severe pain | 0.74 | 0.21 – 1.27 | 0.11 | 0.03 – 0.19 | 0.006* |
| Aggravation by routine physical activity | -0.23 | -0.79 – 0.33 | -0.03 | -0.10 – 0.04 | 0.420 |
| Nausea or vomiting | 1.68 | 1.19 – 2.17 | 0.29 | 0.20 – 0.37 | < 0.001* |
| Photophobia | 1.29 | 0.82 – 1.76 | 0.19 | 0.12 – 0.26 | < 0.001* |
| Phonophobia | 1.19 | 0.70 – 1.68 | 0.21 | 0.12 – 0.30 | < 0.001* |
| Osmophobia | 1.78 | 1.21 – 2.34 | 0.23 | 0.16 – 0.30 | < 0.001* |
| AMD (day/month) | 0.03 | -0.02 – 0.09 | 0.06 | -0.04 – 0.18 | 0.246 |
| Use of prophylactic medication | -0.09 | -0.87 – 0.68 | -0.01 | -0.09 – 0.08 | 0.814 |

This table presents the results of a multivariable linear regression analysis examining which headache characteristics were associated with MIBS-4 score. All explanatory variables were entered simultaneously using the forced-entry method. P-values less than 0.05 were considered statistically significant and are marked with an asterisk (*). The linear regression model demonstrated acceptable explanatory power, with an adjusted R² of 0.302. MHD: monthly headache days; AMD: acute medication days. There are no missing values.

**Supplementary Table 9 Multivariable Linear Regression Analysis of Headache Characteristics Associated with Headache Impact Test-6 (HIT-6) Score among Female Participants**

| Variables | Unstandardized coefficient (B) | 95% confidence interval for B | Standardized coefficient (β) | 95% confidence interval for β | p values |
| --- | --- | --- | --- | --- | --- |
| Age (years) | -0.26 | -0.36 – -0.17 | -0.16 | -0.22 – -0.10 | < 0.001* |
| Duration of headache (h) | 0.04 | 0.02 – 0.07 | 0.11 | 0.06 – 0.19 | 0.001* |
| MHD (days/month) | 0.18 | 0.03 – 0.32 | 0.12 | 0.02 – 0.21 | 0.019* |
| Unilateral pain | -0.31 | -1.28 – 0.65 | -0.03 | -0.12 – 0.06 | 0.529 |
| Pulsating pain | -0.65 | -1.68 – 0.39 | -0.02 | -0.05 – 0.01 | 0.218 |
| Moderate or severe pain | 4.88 | 3.52 – 6.23 | 0.24 | 0.17 – 0.31 | < 0.001* |
| Aggravation by routine physical activity | 1.62 | 0.18 – 3.06 | 0.07 | 0.01 – 0.13 | 0.028* |
| Nausea or vomiting | 2.10 | 0.85 – 3.35 | 0.12 | 0.05 – 0.19 | 0.001* |
| Photophobia | 4.46 | 3.24 – 5.69 | 0.25 | 0.18 – 0.31 | < 0.001* |
| Phonophobia | 0.44 | -0.82 – 1.70 | 0.03 | -0.06 – 0.11 | 0.491 |
| Osmophobia | 2.67 | 1.23 – 4.11 | 0.14 | 0.06 – 0.21 | < 0.001* |
| AMD (day/month) | 0.09 | -0.47 – 0.24 | 0.05 | -0.26 – 0.13 | 0.187 |
| Use of prophylactic medication | -1.26 | -3.25 – 0.73 | -0.02 | -0.05 – 0.01 | 0.215 |

This table presents the results of a multivariable linear regression analysis examining which headache characteristics were associated with HIT-6 score. All explanatory variables were entered simultaneously using the forced-entry method. P-values less than 0.05 were considered statistically significant and are marked with an asterisk (*). The linear regression model demonstrated acceptable explanatory power, with an adjusted R² of 0.368. MHD: monthly headache days; AMD: acute medication days. There are no missing values.

**Supplementary Table 10 Multivariable Linear Regression Analysis of Headache Characteristics Associated with Overall Work Productivity Impairment (OWPI) among Female Participants**

| Variables | Unstandardized coefficient (B) | 95% confidence interval for B | Standardized coefficient (β) | 95% confidence interval for β | p values |
| --- | --- | --- | --- | --- | --- |
| Age (years) | -0.75 | -1.33 - -0.17 | -0.10 | -0.18 – -0.02 | 0.012* |
| Duration of headache (h) | -0.12 | -0.27 - 0.04 | -0.06 | -0.14 – 0.02 | 0.141 |
| MHD (days/month) | 0.15 | -0.69 - 0.98 | -0.19 | -1.24 – 0.87 | 0.735 |
| Unilateral pain | 0.47 | -5.04 – 5.99 | 0.06 | -0.64 – 0.77 | 0.865 |
| Pulsating pain | 0.09 | -5.79 – 5.98 | 0.01 | -0.64 – 0.66 | 0.974 |
| Moderate or severe pain | -4.06 | -12.17 – 3.98 | -0.04 | -0.12 – 0.04 | 0.322 |
| Aggravation by routine physical activity | -0.89 | -9.18 – 7.42 | -0.01 | -0.10 – 0.08 | 0.835 |
| Nausea or vomiting | -8.45 | -15.83 – -1.05 | -0.11 | -0.20 – -0.02 | 0.025* |
| Photophobia | 7.16 | -0.97 – 14.42 | 0.08 | -0.01 – 0.16 | 0.053 |
| Phonophobia | -14.86 | -22.20 - -7.51 | -0.19 | -0.28 – 0.10 | < 0.001* |
| Osmophobia | 6.10 | -2.37 – 14.56 | 0.06 | -0.02 – -0.14 | 0.158 |
| AMD (day/month) | 0.69 | -0.13 - 1.58 | 0.09 | -0.02 – 0.21 | 0.100 |
| Use of prophylactic medication | 4.19 | -7.19 – 15.57 | 0.03 | -0.05 – 0.11 | 0.470 |
| MIBS-4 score (sum) | 3.44 | 2.05 - 4.83 | 0.26 | 0.15 – 0.37 | < 0.001* |
| HIT-6 score (sum) | 0.29 | -0.24 - 0.83 | 0.06 | -0.05 – 0.17 | 0.282 |

This table presents the results of a multivariable linear regression analysis with 1,000 bootstrap samples examining whether MIBS-4 and HIT-6 scores were associated with OWPI. All explanatory variables were entered simultaneously using the forced-entry method. P-values less than 0.05 were considered statistically significant and are marked with an asterisk (*). The linear regression model demonstrated acceptable explanatory power, with an adjusted R² of 0.139. HIT-6: Headache Impact Test-6; MIBS-4: Migraine Interictal Burden Scale. There are no missing values.
